# Research delivery secondments: A scoping review

**DOI:** 10.1002/nop2.2089

**Published:** 2024-01-15

**Authors:** Naomi Hare, Sharon Grieve, Janine Valentine, Julie Menzies

**Affiliations:** ^1^ Guy's and St Thomas' NHS Foundation Trust, St Thomas' Hospital London UK; ^2^ Royal United Hospitals Bath NHS Foundation Trust Bath UK; ^3^ University of the West of England Bristol UK; ^4^ Yeovil Hospital NHS Foundation Trust Yeovil UK; ^5^ University of Bournemouth Poole UK; ^6^ Birmingham Women's and Children's NHS Foundation Trust; Institute of Clinical Sciences University of Birmingham Birmingham UK

**Keywords:** allied health professionals, capacity building, midwifes, nurses, professional practice, research, secondment

## Abstract

**Aim:**

To explore and summarise published literature with regards to secondments to clinical research and to identify the gaps in research to inform further work.

**Design:**

Systematic scoping review.

**Method:**

A scoping review was undertaken in accordance with the Patterns, Advances, Gaps, Evidence and Research framework. Databases searched included CINAHL, PubMed, Medline and Embase. Inclusion/exclusion criteria were applied by two independent reviewers. Two reviewers independently retrieved full‐text studies for inclusion and applied the framework as a tool for synthesising Patterns, Advances, Gaps, Evidence and Research recommendations.

**Results:**

Six papers and one abstract published between 2003 and 2018 were included. All secondees (*n* = 34) were released from NHS posts, with secondments (where specified) ranging in duration from 0.25 to 2 years and for 40%–100% of their working hours. All seven papers reported benefits for personal and professional development, predominantly in the form of personal reflections. Few described involvement with research delivery teams.

**Conclusion:**

Published initiatives vary in nature and lack standardised reporting and measurement of impact. Further research is required to identify benefits at a departmental or organisational level, the facilitators for setting up secondments and the application of knowledge gained from secondment opportunities.

**Implications for the Profession:**

Undertaking a research secondment is reported to offer professional and personal benefit for clinical staff. Research secondments are one way in which a research culture can practically be embedded within clinical settings.

**Impact:**

This scoping review identified a lack of published empirical research seeking to understand research secondments as a tool to enhance research and evidence engagement. Although there is a suggestion that secondments could positively impact staff retention, there is limited evidence about the benefit for the organisation or for patient care. These findings have implications for staff, managers and their organisations.

**Reporting Method:**

The PRISMA‐ScR guidelines were used to guide reporting.

**No Patient or Public Contribution:**

This was not relevant to the research design.

## 1 INTRODUCTION

Key to the success of embedding clinical research within organisational and departmental culture is the development and retention of clinical staff who are committed to the importance of research and innovation within their workplace (Cowley et al., 2020). A robust evidence base demonstrates that there are greater treatment opportunities and improved patient outcomes within research‐active organisations (Boaz et al., 2015; Carrick‐Sen & Moore, 2019). Patients recognise and benefit from the value of research in their care and available treatment options (Jonker et al., 2020; Sacristán et al., 2016). Recognising this value, the Health and Social Care Act 2022 now includes embedding a research‐active NHS as a statutory requirement (UK Government, 2022).

In the United Kingdom, a range of local and national initiatives have provided investment specifically focused on the development of research training and career opportunities for nurses and midwives (Bramley et al., 2018; Castro‐Sánchez et al., 2020; Olive et al., 2022). These initiatives have frequently focused on the development and provision of schemes to support clinical academic career pathways (health care professionals working across both clinical and academic settings to develop and disseminate high‐quality research).

Anecdotally, the authors were aware of a range of research and secondment initiatives for clinical nurses and midwives; however, it was unclear how widespread the introduction of these had been. This scoping review was timely to explore how such roles are defined, organised, implemented and evaluated, to support the future development of these initiatives.

## 2 BACKGROUND

There is growing recognition that programmes and resources that support front‐line clinical staff to expand and develop their research and leadership capabilities are needed. These can facilitate a greater understanding of clinical research delivery and enable participants to lead research‐based practice within their clinical area (Bramley et al., 2018). Indeed, the COVID‐19 pandemic stimulated recognition of the value of research and that of research nurses, as well as the importance of integrating research into everyday practice (Faulkner‐Gurstein et al., 2022; Iles‐Smith et al., 2020; Whitehouse, Harris, et al., 2022). With improved relationships and mutual appreciation between research and clinical staff, it is timely to review what is known about schemes to support exposure and engagement with research delivery.

Secondments can be defined as an employee temporarily changing job role within the same organisation or transferring to another organisation on a full‐time or part‐time basis (Gerrish & Piercy, 2014). Secondments are used widely and successfully within the nursing, midwifery and allied health professional (NMAHP) professions to facilitate short‐term cover of vacant positions and to provide opportunity for the development of specialist skills and knowledge development and translation (Gerrish & Piercy, 2014; Jenkins & Anstey, 2017). They are predominantly used as a vehicle for building clinical, education, audit, teaching or research capacity (Dryden & Rice, 2008; Grindell, 2019; Hamilton & Wilkie, 2001; Richardson et al., 2007).

Within this scoping review, we describe the breadth and depth of existing literature around the topic of secondments within clinical research.

## 3 AIMS

The aim of this scoping review was to explore and summarise published literature with regards to secondments to clinical research, and to identify the gaps in research to inform further work.

The key objectives were to:

- Understand the benefits of research secondments
- Identify the barriers and facilitators to establishing and sustaining research secondments
- Identify common enablers such as funding, job descriptions, competencies and evaluation of secondment roles, schemes or programmes

## 4 METHODS

### 4.1 Design

A scoping methodology provided the opportunity to identify gaps in the research and identify a focus for further work (Arksey & O'Malley, 2005). The Patterns, Advances, Gaps, Evidence for practice and Research recommendations (PAGER) framework was used to report the scoping review findings in a methodologically rigorous manner (Bradbury‐Jones et al., 2021).

### 4.2 Search methods

A comprehensive search of the literature reporting research secondments or placements for nurses and midwives was undertaken in October 2021. A systematic search was conducted of the following electronic databases: CINAHL, PubMed, Medline and Embase. The search terms comprised:

1. Secondment OR placement AND research AND Nurs* OR Midwi*
2. Research AND secondment OR placement
3. Research secondment

Search terms were informed by the key word ‘research secondment’ and its synonyms.

### 4.3 Inclusion criteria

The following inclusion criteria were applied to the search results:

1. Journal articles were identified from 2001 until 2021.
2. Published abstracts and full‐text articles published in the English language
3. The secondment was undertaken by a registered nurse, registered midwife, allied health professional or those in nursing support roles.

There were no restrictions on the location of the study or the design of the secondment, as the intention was to capture all variations in design and setup. The inclusion criteria were applied using the limiters available on the database where possible, and then applied to the title and abstract. Journal articles were identified from 2001 until 2021 to ensure the breadth of historical literature across a range of professions. This date reflects the establishment of the National Cancer Research Network in 2001 and, subsequently, the National Institute for Health and Social Care Research (NIHR) Clinical Research Networks (2007). Both of which formalised the national infrastructure and funding for clinical research delivery across England.

As the focus of this review was secondments designed to promote learning around the delivery of clinical research, articles relating to a research internship, clinical academic pathways or an undergraduate placement were excluded. For the purposes of this scoping review, the NIHR definition of research internships as short‐duration awards delivering taught and academically supervised components supported their exclusion (NIHR, 2023). Articles describing research internships were excluded after appraising the information available in the abstract or, where necessary, the full text. Secondments that were not research‐related were also excluded as they did not fulfil the clinical research criteria. The initial population of interest was nurses and midwives; however, the initial search identified relevant literature related to Allied Health Professionals (AHPs). These papers were eligible to be included as they broadened our understanding of secondments and enabled the findings to be applied more broadly. Secondments related to medicine or dentistry were excluded.

### 4.4 Search outcome

**FIGURE 1:**
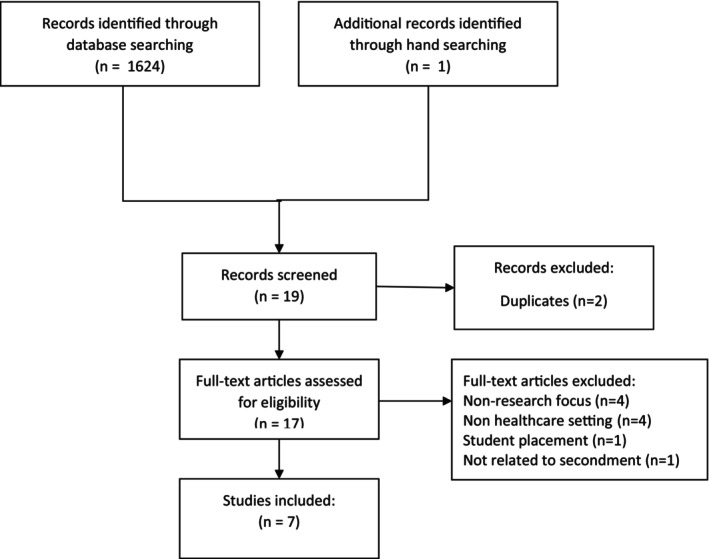
Search flow.

The search results were exported to Rayyan, a web‐based platform that enables collaborative reviews (https://www.rayyan.ai/). Using the Rayyan functionality, the results were reviewed independently by NH and SG. The title and abstract of each paper was examined by either NH or SG and the inclusion and exclusion criteria applied, until all papers had been reviewed. NH & SG classified each paper as include, maybe or exclude. Rayyan has the facility to add notes to facilitate communication between reviewers. Those papers categorised as ‘include’ or ‘maybe’ were then divided between the four members of the research team, the full text reviewed, inclusions discussed and consensus reached. See summary, Figure 1.

### 4.5 Data extraction

All authors extracted data from publications included in the scoping review using a data collection tool developed for the purpose of this review. NH and JM then applied the PAGER framework systematically to the full‐text publications to be included in the review. To facilitate methodological transparency, the PRISMA‐ ScR guidelines (Tricco et al., 2018) were used to guide reporting.

### 4.6 Data synthesis

A literature matrix was created, which was distinct from the data collection tool. The matrix was used to extract the following data from each of the included articles: author, year, title, location of the study, research method, sample size, sample demographic details about the secondment type, duration, specialty, outcomes as well as barriers, benefits and facilitators to the secondment. Outcomes of secondments included any new learning, knowledge, skills or experience gained during or attributed directly to the secondment for the secondee, the manager and the supervisor/research team. Barriers, benefits and facilitators to secondments were similarly reported within the papers, reflecting the three perspectives. Analysis was completed using the PAGER Framework (Bradbury‐Jones et al., 2021) beginning with the identification of patterns to provide a visual representation of the main themes within the literature. This was followed by identifying the contribution of the literature to advancing knowledge, what had been left out of research to date and avenues for further enquiry and recommendations for future research. This was conducted independently by two researchers (NH and JM), who then collaborated to agree upon the final version of the table. Consideration of the evidence for practice identified practical messages extractable from the literature in the form of implications for key stakeholders who may benefit from the findings and implications for the field of knowledge. Finally, research recommendations could emerge that build on the identification of gaps and complement the reporting of the evidence for practice.

## 5 RESULTS

### 5.1 Characteristics of included studies

The scoping review identified seven papers published between 2003 and 2018, all reflecting schemes within the United Kingdom (Table 1). The articles were published in a wide range of journals, from speciality journals such as Paediatric Critical Care Medicine to those designed for a wider audience, for example Nursing Standard. Most of the publications were case studies or personal reflections, with six published articles and one published abstract. The papers reflected the secondment experiences of a range of health care professionals (HCP) (*n* = 34), including nurses (16), physiotherapists (11), occupational therapists (OT) (4), speech and language therapists (SLT) (1), health care assistant (1), and unidentified (1). All secondees were released from NHS posts, with secondment placements ranging from research delivery teams within the same organisation (*n* = 1) (Evans et al., 2018) to research assistant posts under the supervision of a university‐based supervisor/academic unit (*n* = 6) (Daniels & Gospill, 2005; Donaldson, 2011; Loughlin, 2013; Pender, 2011; Pomeroy et al., 2003; Rose & Tuffrey‐Wijne, 2017). The duration of secondment was not specified by three papers (Evans et al., 2018; Loughlin, 2013; Pender, 2011), but within the other four papers there was wide variation, ranging from 0.25 to 2 years. The secondments ranged from a scheme that seconded the individual into research for 100% of their time to schemes that offered between 40% and 80%. The number of secondees reflected in the publications ranged from 1 to 19 (mean 4, median 1). In the publications, secondees were recruited through nomination by an NHS Manager (*n* = 1), responded to an advert (4), unspecified (1), approached a university (1). There was also variety in how the secondments were funded, ranging from specific project grants (2), NIHR/Department of Health schemes (3), to two schemes that did not specify the method (2).

**TABLE 1:** Characteristics of selected studies.

Journal	Study location	No. secondees	Length of secondment (years)	Career stage	Eligibility (occupation)	Appointed (occupation)	Split research:Other role	Substantive organisation	Type of organisation seconded to	Type of secondment	Recruitment method	Funding	Evaluation method
Physiotherapy	North West (NW) England	19	1	Not specified	Therapists and Nurses	Therapists and Nurses	40:60	NHS organisations in NW region	Stroke Association Research Unit	NHS research with Academic/University supervision	Nominated by NHS managers	NW Regional Research and Development directorate	Survey
Therapy weekly	Assistive Technology Centre, Derby, UK	1	1.5	Not specified	Not specified	Physiotherapist	100:zero	NHS organisations in NW region	Assistive Technology Centre, with academic support University of Nottingham	NHS research with Academic/University supervision	Advert in a professional journal	Unclear	Personal reflection
Nursing Standard	York, UK	1	Not specified	Matron	Nurse	Nurse	80:20	NHS organisation	National Institute of Health Research (NIHR)	NHS research with Academic/University supervision	Approached higher education institute to discuss a project and then applied for funding	Service Delivery and Organisation funding University of York	Not specified
Nursing Management	Edinburgh, UK	1	2	Nurse Manager	Not specified	Nurse	50:50	NHS organisation	Research Group, University of Edinburgh	NHS research with Academic/University supervision	Applied for an advertised secondment	Government funded secondment to Primary Palliative Care Research Group, University of Edinburgh	Personal reflection
British Journal of Healthcare assistants	Lancashire Teaching Hospital, UK	1	Not specified	Health Care Assistant (HCA)	Any level of staff'	HCA	40:60	NHS Organisation	University of Central Lancashire	NHS research with Academic/University supervision	Applied for an advertised secondment	NIHR Flexibility and Sustainability funds	Personal reflection
Learning Disability Practice	London, UK	1	0.25	Band 5 RN	Nurse	Nurse	60:40	Community NHS Trust	University of London	NHS research with Academic/University supervision	Applied for an advertised secondment University of London	University of London	Personal reflection
Paediatric Critical Care Medicine	Birmingham, UK	10	Not specified	Band 5 RN	Nurse	Nurse	Not specified	NHS organisation	NHS organisations	Research delivery team within the same NHS Organisation	Not specified	Not specified	Survey

### 5.2 PAGER approach

#### 5.2.1 Patterns

Professional occupation, the number of secondees, the type of organisation seconding staff and the nature of the secondment were well reported across all the included papers (Table 2). There was, however, variation in the reporting of the secondment duration and the division of time between the secondment and the existing role. The gender and career stage of the secondee were well reported, but NHS pay band and level of training were less well documented. The majority of the publications (*n* = 5) were personal reflections of secondee experience, with two papers reporting feedback obtained by questionnaire. Opportunities are predominantly related to staff participation within in a clinical academic collaborative project affiliated to a Higher Education Institution (HEIs) with supervision by academic staff, with only one account describing a secondment to a clinical research delivery team.

**TABLE 2:** Patterns of reporting across the included studies.

Author	Sociodemographic factors						Socioeconomic factors			Publication type		Nature of intervention					Outcomes			Barriers		Benefits			Facilitators to secondments		
	Age	Gender	Career stage	No. Secondees	Occupation	Level of Training	NHS pay banding	Type of Organisation	Employment	Personal reflection	Survey	Location of Secondment	Length of secondment	Whole time equivalent	Supervision	Outline of Intervention/JD	Secondee experience	Manager /team Experience	Supervisor Experience	Management	Secondee	Management	Secondee	Researchers	Managers	Secondee	Researchers
Pomeroy et al. (2003)				X	X			X			X	X	X	X	X	X	X				X		X	X	X	X	
Daniels and Gospill (2005)		X		X	X	X		X	X	X		X	X		X	X	X					X	X				
Pender (2011)		X	X	X	X			X	X	X		X		X	X		X					X	X	X			
Donaldson (2011)		X	X	X	X			X	X	X		X	X	X	X	X	X				X	X	X			X	
Loughlin (2013)		X	X	X	X			X	X	X		X		X	X	X	X				X	X	X	X		X	
Rose and Tuffrey‐Wijne (2017)		X	X	X	X	X	X	X	X	X		X	X	X	X	X	X		X	X	X	X	X	X		X	
Evans et al. (2018)			X	X	X		X	X	X		X	X					X				X		X				

All seven papers reported outcomes related to the secondee, with one publication also including the reflections of an academic supervisor. No papers included feedback from managers or featured the NHS organisational perspective on the secondment. Similarly, barriers to secondments were predominantly reported in relation to the experience of the secondee. Only one paper offered insights into challenges for NHS managers, and these were reflections from the secondee rather than the managers themselves. Benefits for secondees, managers and researchers were better described; however, the accounts were lacking in detail about facilitators to developing and implementing these opportunities. Themes in relation to barriers, benefits and facilitators are reviewed in Table 3. Barriers experienced during research secondments were not widely reported.

**TABLE 3:** Summary of barriers and facilitators for managers, secondees and researchers identified within the published literature.

Staff group	Barriers	Reference(s)	Benefits	Reference(s)	Facilitators to secondment	Reference(s)
Managers	Level of supervision	Rose and Tuffrey‐Wijne (2017)	Promote evidence based practice	Pomeroy et al. (2003), Daniels and Gospill (2005), Donaldson (2011), Pender (2011), Loughlin (2013)	Adapt to feedback	Pomeroy et al. (2003)
	Level of supervision Competing demands	Rose and Tuffrey‐Wijne (2017), Pomeroy et al. (2003), Donaldson (2011), Rose and Tuffrey‐Wijne (2017)	Service development	Pomeroy et al. (2003), Donaldson (2011), Daniels and Gospill (2005), Rose and Tuffrey‐Wijne (2017), Evans et al. (2018)	Fill short term positions	Loughlin (2013)
			Staff professional development	Rose and Tuffrey‐Wijne (2017)	Right balance of roles	Pomeroy et al. (2003)
			Staff professional development Personal development	Rose and Tuffrey‐Wijne (2017), Pomeroy et al. (2003), Daniels and Gospill (2005), Pender (2011), Loughlin (2013), Rose and Tuffrey‐Wijne (2017), Evans et al. (2018)	Vision for secondee	Pomeroy et al. (2003)
					Supervision	Pomeroy et al. (2003), Loughlin (2013), Rose and Tuffrey‐Wijne (2017)
Secondees	Lack of knowledge and skills	Pomeroy et al. (2003), Daniels and Gospill (2005), Donaldson (2011), Loughlin (2013)	Development of others	Pomeroy et al. (2003), Daniels and Gospill (2005), Donaldson (2011)	Collaboration and Multi‐disciplinary team involvement	Pomeroy et al. (2003), Loughlin (2013)
	Human Resources /Payroll	Rose and Tuffrey‐Wijne (2017)	Staff retention	Rose and Tuffrey‐Wijne (2017), Evans et al. (2018)	Training and personal development	Pomeroy et al., (2003), Rose & Tuffrey‐Wijne, (2017)
	Organisational culture	Pomeroy et al. (2003)	Patient care	Loughlin (2013)	Appropriate attitude	Pomeroy et al., (2003), Rose & Tuffrey‐Wijne, (2017)
	Lack of support	Pomeroy et al. (2003)			Clinical skills and experience	Pomeroy et al. (2003), Rose and Tuffrey‐Wijne (2017)
	Lack of resources	Pomeroy et al. (2003)			Appropriate training	Pomeroy et al. (2003)
	Regulatory approvals	Rose and Tuffrey‐Wijne (2017)			Funding	Pomeroy et al. (2003), Loughlin (2013), Rose and Tuffrey‐Wijne (2017)
	Post secondment	Pomeroy et al. (2003)			Management buy in	Pomeroy et al. (2003), Loughlin (2013)
	Inequality	Evans et al. (2018)			HR processes	Rose and Tuffrey‐Wijne (2017)
		Evans et al. (2018)			Previous roles/experience	Rose and Tuffrey‐Wijne (2017)
					Understanding of culture	Pomeroy et al. (2003)
			Awareness of NHS research	Pomeroy et al. (2003), Donaldson (2011), Pender (2011)		
Researchers			Facilitate implementation into practice	Pomeroy et al. (2003), Pender (2011), Loughlin (2013)		
			Connection to ‘real world’ practice	Pomeroy et al. (2003), Rose and Tuffrey‐Wijne (2017)		
			Stimulate post‐grad research	Pomeroy et al. (2003)		
			Support grant writing	Pomeroy et al. (2003)		
			Networking	Pomeroy et al. (2003)		

The main challenges reported by secondees were managing competing demands or juggling two roles (Donaldson, 2011; Pomeroy et al., 2003; Rose & Tuffrey‐Wijne, 2017) and initially lacking sufficient knowledge and skills (Daniels & Gospill, 2005; Donaldson, 2011; Loughlin, 2013; Pomeroy et al., 2003). All seven papers reported on the benefits of the secondments for staff personal and professional development. For some personnel, this enhanced the way they managed others (Daniels & Gospill, 2005; Donaldson, 2011; Pomeroy et al., 2003). Other reported benefits included staff retention (Evans et al., 2018; Rose & Tuffrey‐Wijne, 2017) and enhanced patient care (Loughlin, 2013). Secondees referred to perceived benefits for managers/services, in the form of enhanced service development (Daniels & Gospill, 2005; Donaldson, 2011; Evans et al., 2018; Pomeroy et al., 2003; Rose & Tuffrey‐Wijne, 2017) and evidence‐based practice (Daniels & Gospill, 2005; Loughlin, 2013; Pender, 2011; Pomeroy et al., 2003; Rose & Tuffrey‐Wijne, 2017). However, no papers included the perspective of the managers themselves.

Facilitators for setting up and successfully supporting a secondment were not widely reported in the papers. Reflections included the need for managers to have a vision for secondees on completion of the opportunity (Pomeroy et al., 2003), the need to respond to secondee feedback to shape and improve secondments (Pomeroy et al., 2003), to offer flexibility to balance other roles (Pomeroy et al., 2003) and to fill short‐term positions (Loughlin, 2013). For secondees, the key facilitators were adequate and appropriate supervision (Loughlin, 2013; Pomeroy et al., 2003; Rose & Tuffrey‐Wijne, 2017) and designated funding (Loughlin, 2013; Pomeroy et al., 2003; Rose & Tuffrey‐Wijne, 2017). There was also recognition of the secondee being at the right point of their professional training (Pomeroy et al., 2003; Rose & Tuffrey‐Wijne, 2017), with the right knowledge and skills (Pomeroy et al., 2003; Rose & Tuffrey‐Wijne, 2017) and attitude (Pomeroy et al., 2003; Rose & Tuffrey‐Wijne, 2017) and the importance of collaboration (Loughlin, 2013; Pomeroy et al., 2003) and training opportunities (Pomeroy et al., 2003; Rose & Tuffrey‐Wijne, 2017).

#### 5.2.2 Advances

The articles describe a wide variety of creative opportunities set up between the NHS and academic partners across a range of health professions and organisations. They demonstrate that initiatives to increase nursing and allied health professional's ability to actively engage in and support clinical research have been in existence for almost 20 years. Secondment opportunities offer professional development for the secondee and may support and guide the professional development of others. Despite the history of these types of opportunities, knowledge and evidence surrounding secondments have not advanced, remaining as reflective accounts or self‐reported outcomes in non‐validated questionnaires.

#### 5.2.3 Gaps

There is a lack of empirical evidence about the role and value of secondments to research. Secondees report favourably about these opportunities, particularly for their personal development, but there is little evidence about the value for their NHS Managers and their employing organisation. There is also a gap in reporting about the challenges of these opportunities from the perspective of all stakeholders.

Secondments varied significantly in duration, length and content. This lack of standardisation about what a secondment is, or entails, creates challenges for summarising the benefits or identifying who could most benefit from such opportunities. The published literature also fails to report on the academic qualification of secondees and their clinical or research development following the secondment. Only one of the included papers described a secondment to a research delivery team and the secondees role in research delivery, which highlights a gap about the value of these type of opportunities.

#### 5.2.4 Evidence for practice

The published literature provides some evidence that secondments can positively influence the personal and professional development of individuals, which is important information for NHS managers, when considering staff recruitment and retention (Table 4). However, the lack of published accounts from managers and researchers creates challenges for making the case that these opportunities add value to service provision, and future evaluation needs to be more standardised to capture the impact of undertaking a secondment. The variation in reporting also needs to improve to articulate where secondments run, how they are funded, what makes them work and what impact they can have on services. This will also enable services to plan how opportunities can be sustained and increased to benefit more staff. Five of the seven accounts currently only detail an opportunity for one person, with no indication about whether there were subsequent schemes.

**TABLE 4:** Summary of the evidence.

	Pattern	Advances	Gaps	Evidence for practice	Research recommendations
1	Majority of the evidence is personal reflections, not empirical research	Staff seconded to research roles perceive themselves to have benefitted professionally	Low levels of evidence measuring professional and personal benefit for the secondees	Evidence that secondments can positively influence personal development of individuals; important for staff recruitment and retention and managers	Further qualitative research is required to understand the value and impact of secondments from the secondee perspective
2	Published literature have predominantly focused on outcomes related to the secondee	Value of research secondments for secondee personal development and research capacity skills is articulated	Lack of research addressing the perspective of managers and researchers on the impact and value of secondments for staff Low level of evidence evaluating the benefits of research secondments for patient care and service provision	NHS Managers may benefit from signposting to how secondment posts can be created and the value of research secondment posts Low‐level evidence that research secondments promote evidence‐based care and service development	Further research is required to understand the benefits and challenges of seconding staff for the individual, the department and the organisation
3	Wide variety of research secondments have been created for a wide range of NHS health care professionals	Wide variety of opportunities have been established between NHS and academic partners, across a range of health professions and types of organisations	Lack of standardisation about what a secondment entails creates challenges for summarising the benefits Lack of information about academic qualification of secondees and clinical or research development following secondment	Need improved links between HEI settings to enhance recruitment of NHS staff to research assistant posts and projects which meet personal and organisational research priorities	Further research is required to explore the barriers and facilitators to joint working between the NHS and HEI/academic unit
4	Limited reporting about facilitators for setting up and coordinating secondments	Research secondments benefit from training, supervision and management engagement	Lack of evidence about the best methods to set up/support research secondments Lack of perspective from managers and organisation about facilitating secondment opportunities	Need to articulate where secondments run, how they are funded, what makes them work and what impact for services they can have Expansion is required about how opportunities are created and sustained	Research needs to explore barriers and facilitators to research secondments at the department and organisational level
5	Opportunities predominantly relate to staff engagement in clinical academic opportunities with HEI & academic supervision	Opportunities being created to increase NMAHP ability to actively engage and support clinical research	Only one paper describes a secondment to a research delivery team and their role in research	Exposure to research delivery teams can help recruitment to research posts, enhance understanding of NHS research and contribute to a positive research culture	Further research is required to offer opportunities for NHS staff to engage with research delivery teams

#### 5.2.5 Research recommendations

There have been a wide variety of secondments created and offered to a range of NHS health care professionals. However, it was a complex task to summarise the value of staff secondments as they occurred across different settings with different methods of supervision and a lack of standardised reporting about the expected outcomes and measurement of impact. Although the secondee perspective has been captured from 34 participants, further research is required to understand the benefits of seconding staff for their department and organisation, the facilitators for the creation and co‐ordination of secondments and the application and impact of knowledge gained from such opportunities. Further research is required to understand why there are few accounts of secondment to clinical research delivery teams and identify barriers and facilitators to joint working between the NHS and HEI/academic units.

## 6 DISCUSSION

This scoping review has comprehensively mapped the available literature pertaining to research secondments for NMAHP's. The literature, whilst limited in its breadth, demonstrates the value of research secondments predominantly to the individual, with some suggestion of the wider benefits to patients, departments and organisations. Embedding a research culture remains a challenge despite concerted effort and statutory footings, but our findings demonstrate that research secondments could be one tool to ensure NMAHPs develop practical research skills.

Research secondments have the potential benefit of accelerating the research capability of the clinical workforce by providing practical experience and providing a first step into clinical academia or research delivery careers (Evans et al., 2018; Richardson et al., 2007). This is reflected in the recently published Chief Nursing Officer for England's strategic plan for research (NHSEI, 2021), which calls for the greater involvement of all nurses in research, with increased opportunities to develop research knowledge and skills applicable to all areas of practice. Only one paper describes the secondment of clinical staff to research delivery teams that are situated within clinical arena's. Whilst there were clear benefits to secondments within academic settings, the authors postulate that increasing short‐term secondment opportunities for clinical research teams (within their own department or organisation) will: improve understanding of research in practice; improve awareness of clinical research nurse, midwife or AHP roles; and have longer term benefits for local departments in terms of retention, research awareness and enhanced care from evidence awareness and research confidence.

Although anecdotally there are a range of research and secondment initiatives across the United Kingdom for clinical nurses and midwives, there are no clear models or strategies through which to implement and evaluate such initiatives. Each development forges its own path and often commences without explicit objectives. Whilst there is value in individual accounts and personal stories, there is a lack of empirical research. As a result, our understanding of what works remains limited, and recommendations around key areas such as how to scale up research secondments, standardise their design, identify metrics for success, articulate impact and ensure fair and equitable access to secondment opportunities remain outstanding.

There have been a range of national and local initiatives launched that have provided investment specifically aimed at the development of research training and career opportunities for nurses and midwives (Bramley et al., 2018; Castro‐Sánchez et al., 2020; Manning, 2022; Menzies et al., 2021, 2022; Olive et al., 2022; Pattison et al., 2022; Sanders et al., 2022; Shepherd et al., 2022; Whitehouse, Tinkler, et al., 2022), and it is clear that the secondment of clinical staff to research roles also offers a key opportunity for professional development. We know that professional and career opportunities are central factors in retaining staff within departments or organisations (Carter & Tourangeau, 2012). With an evolving workforce crisis across both the NHS and globally (Royal College of Nursing, 2022; The Kings Fund, 2022), a contemporary topic in healthcare is retaining and motivating experienced staff. The review findings suggest that research secondments could be a method through which to keep experienced staff, kick‐start their research engagement and facilitate research within departments. Clinical secondment opportunities, more generally, raise staff motivation and aid retention (Dryden & Rice, 2008) and are overwhelmingly seen as a worthwhile opportunity, allowing individuals to develop new skills and knowledge, progress their career and gain a broader strategic perspective (Hamilton & Wilkie, 2001). It is important to note that the ongoing support of managers from seconding organisations is required to maximise the benefits to both the individual secondees and their organisation (Gerrish & Piercy, 2014).

## 7 LIMITATIONS

Confusion as to what a research secondment should achieve persists, with wide‐ranging objectives and varying terminology; with terms such as internships, secondments, placements and even fellowships being used to describe such initiatives. Alongside this, there is limited empirical research seeking to understand the value, facilitators and challenges of research secondment opportunities for NMAHPs. The original aim of this scoping review was to identify common enablers such as funding, job descriptions, competencies and evaluation of research secondments. Despite a comprehensive search of the literature, there was a low level of detail in the included papers to report on this well. This may reflect the fact that the accounts were predominantly personal narratives that did not include any objective evaluation that may or may not have been undertaken by the organisation or those arranging secondment opportunities. This might indicate that the further data relating to the outcomes of the secondment, rather than not having been recorded, was simply not within the scope of the paper. It is recognised that the search term ‘allied health professional’ will not have captured the diversity of this group of professions. Future work would benefit from the search being expanded to include those terms describing specific professions within this group.

## 8 CONCLUSION

The secondment of clinical NMAHPs to a research team is beneficial and may be a valuable tool for embedding research culture within a clinical setting and retaining experienced staff. There is a lack of empirical research exploring the utility, benefits and challenges of clinical research within clinical practice. The current evidence is predominantly narrative reviews by a single author, but, despite this, there are suggestions of significant personal and professional benefit. Further research is being undertaken by the authorship following this review, specifically pertaining to the manager's perspective on supporting, funding and developing research secondment opportunities.

### RELEVANCE TO CLINICAL PRACTICE

- Undertaking a research secondment is reported to offer professional and personal benefits for clinical staff. Although there is a suggestion that this could positively impact staff retention, there is limited evidence about the benefit for the organisation or for patient care.
- Regardless of design, research secondments benefit from engaged managers, regular support and exposure to relevant training.
- Research secondments are one way in which a research culture can practically be embedded within clinical settings.
- Further research should focus on the facilitators for setting up, funding and managing research secondments, with the aim of providing evidence to support managers to effectively and efficiently setting up and supporting successful research secondment opportunities.

## AUTHOR CONTRIBUTIONS

Study concept and design: NH, SG, JV, JM. Data curation: NH, SG, JV, JM. Data analysis: NH, SG, JM. Manuscript‐ original draft: NH, SG, JV, JM. Manuscript‐ review and editing: NH, SG, JV, JM.

## FUNDING INFORMATION

No funding was required for this study.

## CONFLICT OF INTEREST STATEMENT

None declared. All authors were National Institute for Health Research (NIHR) 70@70 Senior Nurse and Midwife Research Leaders. The views expressed in this article are those of the authors and not necessarily those of the NIHR.

## Data Availability

Data sharing not applicable – no new data generated.
